# Dataset of urban nature games to aid integrating nature-based solutions in urban planning

**DOI:** 10.1016/j.dib.2023.109800

**Published:** 2023-11-10

**Authors:** Aura-Luciana Istrate, Perrine Hamel

**Affiliations:** a University College Dublin (UCD), School of Architecture, Planning and Environmental Policy, Richview, Clonskeagh, Dublin, D14 E099, Ireland^1^; b Nanyang Technological University (NTU), Asian School of the Environment, 639798, Singapore

**Keywords:** Games database, Gaming, Game-based approaches, Ecosystem services, Nature-based solutions, Urban planning, Urban design

## Abstract

This dataset of Urban Nature Games provides information, ratings, and categorizations of different types of games that incorporate concepts of urban planning and ecosystem services or nature-based solutions. It consists of games retrieved from systematic searches on various search engines and public databases, using keywords related to: urban design and planning; ecosystem services and nature-based solutions; and game-based approaches. Recorded meta information includes game names, developers, links to each game's documentation, relevant publications, as well as generic playing information such as play duration, number of players, target group, distribution format, play mode, and costs. Of the 69 games compiled, 37 games have been rated of “high” to “medium” relevance based on their descriptions, and have been further assessed and categorized based on a framework incorporating concepts of urban planning and nature-based solutions, the game's scope, and practice. Among the 22 “high” relevance games, 41% can and have been used to engage multiple stakeholders, and 36% to engage citizens and communities. This data article relates to the research article entitled “Urban Nature Games for integrating nature-based solutions in urban planning: a review”, and presents a more detailed, editable version of the dataset. The purpose is to provide a hands-on resource for educators, practitioners, and researchers to directly enable them to select their most suitable games linking ecosystem services and nature-based solutions with urban planning.

Specifications Table

**Table:** 

Subject	Planning and Development; Nature and Landscape Conservation;
Specific subject area	Game-based approaches for integrating nature-based solutions in urban planning
Data format	Raw, Analyzed, Filtered
Type of data	Tables (.xls file, dataset with labels, text, and links)
Data collection	Games available in English were compiled from internet searches using various keywords (gam*/gaming/serious gam*/simulation* + urban plan*/urban design/planning + ecosystem service*/nature-based solution*/nature) and systematic searches on available game databases: Gamepedia; Ludoscience; Games for Cities; tabletopia.org; ecogamer.org; and boardgamegeek.com. The games were categorized and assessed following a conceptual framework reflecting i) incorporated concepts of urban planning and ecosystem services/nature-based solutions, ii) the game's scope including their purpose, learning outcomes, and intended impact, and iii) the practice of such games.
Data source location	Data on games were collected to include a global range, by researchers working at two institutions: Institution 1: Nanyang Technological University, The Asian School of the Environment City/Town/Region/Country: Singapore Geographical coordinates: 1°20′53.92″ N; 103°40′59.28″ E Institution 2: University College Dublin, School of Architecture, Planning, and Environmental Policy City/Town/Region/Country: Dublin, Ireland Geographical coordinates: 53°18′23.22″ N; 6°13′07.30″ W Data are stored in the Mendeley repository.
Data accessibility	Repository name: Mendeley Data identification number: 10.17632/2cfbs5gd9t.4 Direct URL to data: https://data.mendeley.com/datasets/2cfbs5gd9t/4
Related research article	Istrate, A.-L., & Hamel, P. (2023). Urban Nature Games for integrating nature-based solutions in urban planning: A review. *Landscape and Urban Planning, 239*, 104860. https://doi.org/10.1016/j.landurbplan.2023.104860

## 1 Value of the Data

• These data are useful in understanding the potential of Urban Nature Games in linking concepts of ecosystem services/nature-based solutions and urban planning.
• Given the systematic collection and assessment process, the dataset provides a unique detailed summary of existing games that can link environmental and urban planning fields;
• Educators, practitioners, and researchers can benefit from this dataset by gaining an overview of available Urban Nature Games with different purposes and potential outcomes, from which to select the most suitable ones according to their needs.
• Researchers and practitioners may investigate the effectiveness of games in better integrating nature-based solutions in urban planning in comparative studies, or develop further games that tackle some of the current shortcomings and limitations.
• The dataset can be a support for future research and practice among the interdisciplinary environmental and planning fields and can help researchers and practitioners develop further hypotheses and queries;
• Data can be further analyzed by performing correlations and identify further patterns and trends in the gaming landscape;
• Researchers and practitioners can further extend the dataset by including AR/VR games, mobile or gamified ‘apps’, or games in different languages.

## 2 Data Description

The dataset consists of a main .xls file containing three spreadsheets that categorize, rate, and describe games based on different dimensions relevant to ecosystem services/nature-based solutions and urban planning. Games in the first spreadsheet (“Categorized 22 High-relev Games”) are a subset of those listed in the second spreadsheet (“Ratings 37 High + Medium Games”), which themselves are a subset of those listed in the last spreadsheet (“All 69 games described”).

**Fig. 1:**
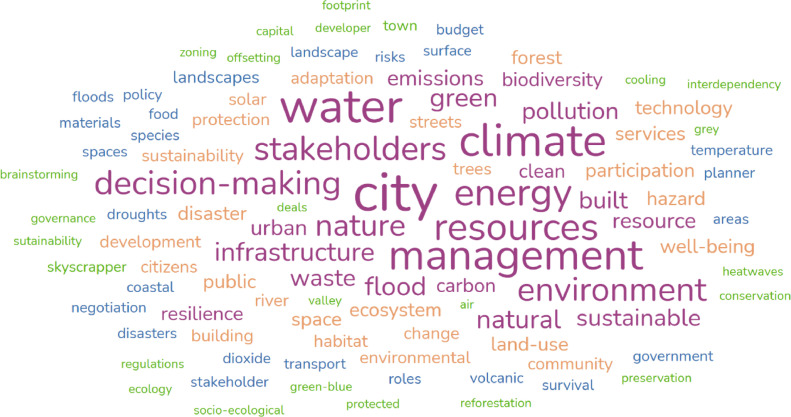
Word cloud of keywords to be used for quick search among all 69 games in the database.

The spreadsheet “All 69 games described” in the .xls file contains short descriptions of the 69 games, along with excerpts of their relevant aspects for 1) urban planning and 2) ecosystem services/nature-based solutions, and their rated relevance for the two fields, from high to low (see details in the methods section of this article). Generic characteristics of games are also included (Table 1), such as the time of play, whether it is a singleplayer or multiplayer game, target group (children, youth, or adults), distribution format (digital or analogue), play mode (online or offline), and costs (with costs or for free). These generic categorizations enable searches for game types in the dataset. Additionally, keywords summarizing the main aspect touched upon in each game are provided (column S of “All 69 games described”), summarized in a word cloud in Fig. 1, to make the dataset more user friendly for quick search. The game developers or publishers, the release/update years, and links to available materials and publications are also provided for each game.

**Table 1:** Generic characteristics of the 22 high-relevance games.

Generic game features	Frequency	Percentage
***Distribution***		
Analog	7	32%
Digital	12	54%
Digital & Analog	3	14%
***Play Mode***		
In-Person	6	27%
Online	8	36%
Online & In-Person	8	36%
***Costs/fees***		
Charges Fee	4	18%
For Free	15	68%
For Free (edu) & Charges Fee	3	14%
***Target Audience***		
Adults	10	45%
Youth	4	18%
Youth & Adults	8	36%
***Players***		
Multiplayer	11	50%
Single Player	4	18%
Single Player & Multiplayer	7	31%
***Time to Play***		
<1h	2	9%
1–2h	4	18%
>2h	5	22%
Varied	4	18%
No info	7	32%

**Fig. 2:**
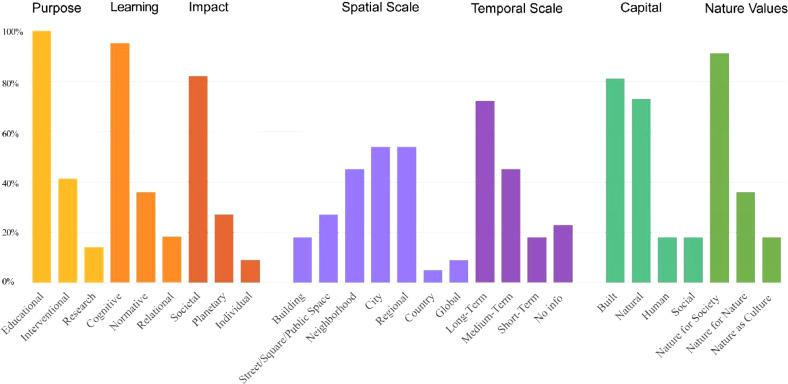
High-relevance games (*n* = 22) categorized according to different dimensions of analysis.

The spreadsheet “Ratings 37 High + Medium Games” in the .xls file provides the categorization of games of high to medium relevance, according to dimensions defined in the conceptual framework [1]. The spatial scale (building; street/square/public space; neighbourhood; city; regional; country; global level), temporal scale of actions proposed through the game (short, mid, or long-term), types of capital promoted through each game (natural, built (i.e. economic), social, and human), the Nature Values elicited through the game as defined in the Urban Nature Futures Framework (UNFF, referring to Nature for Nature (promoting intrinsic values), Nature for Society (promoting instrumental values), and Nature as Culture (promoting relational values)) are dimensions relating to how the games capture important concepts from urban planning and ecosystem services/ nature-based solutions (Fig. 2). Another group of dimensions includes the game's purpose (educational, interventional, or for research), learning (cognitive, normative, or relational), and intended impact (individual, societal, or planetary), representing the game's scope (Fig. 2). One more group of dimensions includes information on the practice of games, such as, whether, at the time of compiling this database, the game provided info on debriefing/dialog to be conducted with the players, whether it includes real data, whether the game has been tested with multiple users, and if there was any pre- or post-game assessment performed. The games’ applicability (Global North, Global South, or general applicability) is also provided. The complexity level of each game was rated considering students in their final highschool years or beginning their undergraduate studies as a reference (Table 2). The definition of each dimension is provided in Table 3.

**Table 2:** The 22 high-relevance games in practice.

Dimensions	Frequency	Percentage
***(De)briefing/Dialogue***		
Yes (in publications)	18	82%
Yes (in game websites/databases)	14	64%
No info in either	4	18%
***Pre/post-game assessment***		
Yes (in publications)	9	41%
Yes (in game websites/databases)	2	9%
No info in either	13	59%
***Tested with multiple user groups***		
Yes (in publications)	10	45%
Yes (in game websites/databases)	4	18%
No info in either	12	55%
***Real Data***		
Yes	10	45%
No	12	55%
***Complexity***		
Balanced	11	50%
Complex	9	41%
Simplified	2	9%

**Table 3:** Defined dimensions of assessment for Urban Nature Games (Source: adapted and expanded from [1]).

Dimensions	Description/definition
***Concepts of ES/NBS and urban planning in games***	
**Urban Planning Processes**	
*Built environment modeling/practice*	The game serves for: envisioning future city development; modeling the built environment in terms of urban form and infrastructure; exploring built environment practices and building scenarios.
*Citizen participation*	The game serves as a tool to engage common citizens.
*Multi-stakeholder decision-making*	The game serves as a tool for stakeholders’ (other than citizens) decision-making, co-creation, or engagement.

**Spatial scale** (refers to the level that is primarily addressed in the game/the spatial context of the NbS solutions in the gameplay)	

*Site Level* *-Building/Lot;* *-Street/ Square/ Public Space* (which often overlap)*;*	Concerning individual parcels/lots/buildings/a street segment/a square or a small public space. NbS types applicable at the site level include green roofs/walls, street trees, bioswales, small green square, small water splash etc.
*Neighborhood & City level* *-Neighborhood;* *-City;*	Extending beyond a site level, concerning entire neighborhoods, districts, or the entire city. NbS types include parks, network of green spaces, mini/urban forests, urban lakes, etc.
*Regional level and above* *-Regional;* *-Country;* *-Global;*	Concerning vast territories from the regional level to the global level. NbS types include forests, green belts, riparian buffers, etc.

**Temporal scale** (it refers primarily to the time considered in the gameplay – e.g., derived from one play round representing 1 year in DisCoord, to 5 years for climate-proofing a city in Climate Smart, to 10 year sessions in Climate Adaptation Game; if this was not clearly specified in the game, it's the time required to implement game decisions, with regards to integrating NbS in urban settings, especially if these latter notions are clearer)	

*Short-term*	A few months, less than 1 year
*Mid-term*	Between 1 and 5 years
*Long-term*	Over 5 years
**Capital** (types of capitals promoted in games)	
*Natural Capital*	Natural resources (including consideration/protection/regeneration of natural resources as part of the game play, such as air, water, soil, living things)
*Social Capital*	Social networks (increasing or enhancing networks and relationships as part of the main results/objectives of the gameplay)
*Human Capital*	Knowledge and skills of people (significantly increasing human knowledge through the game)
*Built Capital*	All that is man-made (includes buildings, transportation, manufactured goods, income, etc.)

**Nature Values** (UNFF, examples adapted from [6])	

*NN*	*Nature for Nature:* e.g., rewilding city parks, restoration of ecosystems, etc.
*NS*	*Nature for Society:* e.g., nature-based solutions implemented to improve health and well-being of human society
*NC*	*Nature as Culture:* e.g., urban gardens and other communitarian spaces meant to promote a cultural experience of nature

***Scope of urban nature games***	

***Purpose***	
*Education*	With primary learning objectives (players learn new notions and expand their understanding)
*Intervention*	Triggers change (in a specific context). This includes games with a learning purpose played with stakeholders that reflect specific decisions they make in real-life.
*Research*	The game is used for data collection (or other research processes)

***Learning***	

*Cognitive*	Acquiring new knowledge and thinking (e.g., learning about sustainability and ES in cities)
*Normative*	Updating norms and approaches or updating players’ mental models with regards to these (e.g., players get a clear understanding of what are the norms/procedures/policies to integrate ES in cities)
*Relational*	Forming new or expanded networks among players (e.g., stakeholders from different domains get to interact and exchange views)
***Intended impact*** (examples adapted from [7])	
*Individual*	Concerning individual benefits (e.g., knowledge of switching to energy efficient solutions in one's own house)
*Societal*	Concerning benefits for the society, beyond the individual level (e.g., consumption awareness for the society to promote energy efficiency in a community)
*Planetary*	Concerning benefits at the level of the planet, (e.g., pro-environmental behaviors towards energy efficiency for reducing the pressure on the planet)

***Urban nature games in practice***	

**Complexity**	
*Simplified*	The game introduces notions of cities and nature on a very basic level
*Complex*	The game makes use of large amounts of data and multiple features in relation to urban planning/cities and ES/NbS/nature
*Balanced*	The game is neither too simple, nor too complex (it is adaptable to different audiences; the game is presented in an accessible manner)

**Applicability**	
*Global South*	Games primarily tested in and addressed to low- and middle-income areas; often politically or culturally marginalized (i.e., subject to social polarization);

*Global North*	Games addressed to high-income areas, with high human development; or depending on high amounts of data, usually not available in the Global South;
*General applicability*	Games that are playable in both Global North and Global South, and not distinguishing between low or high-income areas within the game;

Starting from this database [2], more specific analyses of the 22 high-relevance games for the urban planning field have also been conducted, such as whether the game is useful for built environment modeling and practice (i.e., 63% of the high-relevance games serve for envisioning future development or exploring future built environment scenarios), for stakeholder decision making (i.e., 41% of high-relevance games can serve as a tool for co-creation or engagement of multiple categories of stakeholders), or for citizen participation (i.e., 36% of the high-relevance games can serve as a tool to engage common citizens). These categories, along with whether the game incorporates nature-based solutions at the site level, neighbourhood & city level, or at the regional level and above, are presented in the spreadsheet “Categorized 22 High-relev Games” in the .xls file.

## 3 Experimental Design, Materials and Methods

### 3.1 Compiling the database

**Fig. 3:**
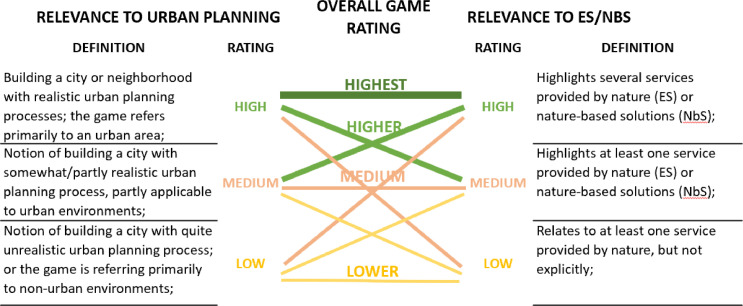
Determining games’ relevance to urban planning and ES/NbS (Source: adapted from [1]).

To compile the database of Urban Nature Games, parallel and snowballed processes were employed using a wide range of sources. Our aim was to be as inclusive as possible when considering the types of games. The main criteria were to include games with purposes other than entertainment (serious games, role-play games, social simulations, board games, etc.) that have relevance to the fields of urban planning and ecosystem services/nature-based solutions. We first conducted systematic searches (between December 2020 and May 2021), in three games databases: the Gamepedia collection of digital and analog games [3]; Ludoscience containing only digital games [4]; and Games for Cities focused particularly on urban games, digital or analog [5]. We supplemented these with internet searches (on the Google and Bing search engines), performed between January 2021 and May 2023 using keywords of (gam*/ gaming/ serious gam*/ simulation*), (urban plan*/ urban design/ planning), and (ecosystem service*/ nature-based solution*/ nature) in various combinations, using Boolean operators AND and OR, for example [serious gam* AND urban plan* AND (ecosystem service* OR nature)]. Other databases we screened include tabletopia.org and ecogamer.org (between August 2021 and May 2022), as well as boardgamegeek.com (between February 2023 and May 2023) to ensure the inclusion of the most relevant games. The games had to be developed by universities, researchers, NGOs, or other credible organizations. Following these searches performed by the main authors of this data article and three additional research assistants (contributors to the database, see [2]), the inclusion of games has been determined based on their relevance to urban planning and nature concepts (see Fig. 3), resulting in a database comprising 69 relevant games, excluding those only conceptualized and not yet available for play, or not available in English. We included both analog and digital games, and among the latter, they had to be accessible to play on Personal Computers (PCs). Games playable only via mobile apps, on specialized platforms such as virtual and augmented reality (VR/AR), or other gamified applications have not been included.

### 3.2 Categorizing and assessing urban nature games

We first rated Urban Nature Games from high to low according to criteria we defined (see Fig. 3). A high relevance to urban planning was attributed when the game referred to an ‘urban’ area and realistic urban planning processes; medium relevance was attributed to games partly applicable to urban environments, with less realistic urban planning processes; low relevance was attributed to games referring primarily to non-urban environments, e.g., virgin islands, and presenting quite unrealistic, e.g., fantasy-derived processes. Similarly, high relevance to ecosystem services/nature-based solutions was attributed to games highlighting several services provided by nature (i.e. ecosystem services) or specific nature-based solutions; medium relevance was attributed to games highlighting at least one service or nature-based solution; low relevance was attributed to games relating to at least one service provided by nature or nature-based solution, but not explicitly. The combined high to low relevance for the two fields determined the overall relevance of games (Fig. 3); for example, those of high relevance to both urban planning and ecosystem services were considered of the ‘highest’ relevance overall.

For those games overall rated of medium to the highest relevance, more extensive game categorization and assessment were conducted, following the defined dimensions (presented in Table 3). These dimensions of analysis were extracted from relevant academic literature on gaming and game-based approaches extracted primarily from the Web of Science and Google Scholar databases (initial searches were systematically performed between December 2020 and January 2021, followed by snowballing and a second round of systematic searches performed between February 2023 and May 2023). The full list of relevant papers is presented in [1]. To assess and categorize the games, information found within game materials, websites, databases, and available publications (subsequently compiled) have been considered (provided in the .xls spreadsheet “All 69 Games described” of the dataset [2]). All games have been reviewed and categorized by two or three contributors, and contrasting results have been solved through discussion.

## Limitations

Including games documented only in English represents a main limitation of our dataset. This limitation is also reflected in whether they can be considered applicable to the Global South, Global North, or have a `general applicability’, which we defined in Table 3 (if considering percentages of the applicability categories, there may be a potential language bias).

The dataset includes multiple types of games (used for purposes other than just entertainment), but it is not exhaustive and can further be added upon as new games become available. Furthermore, the ‘Urban Nature Games’ dataset can be further refined, e.g., structured according to the type of game (i.e. serious games, role-play games, social simulations, browser games, etc.). The dataset can be further developed to also include AR/VR Games, Mobile Games, or Gamified Applications relevant for nature-based solutions and urban planning.

## Ethics Statement

The authors have read and follow the ethical requirements for publication in Data in Brief and confirm that the current work does not involve human subjects, animal experiments, or any data collected from social media platforms.

## CRediT authorship contribution statement

**Aura-Luciana Istrate:** Conceptualization, Methodology, Data curation, Validation, Visualization, Supervision, Writing – original draft, Writing – review & editing. **Perrine Hamel:** Conceptualization, Methodology, Validation, Funding acquisition, Writing – review & editing.

## Data Availability

Urban Nature Games database (Original data) (ICPSR)
